# Functional Recovery Promotion After Spinal Cord Injury With Astaxanthin Treatment in Preclinical Studies: A Systematic Review and Meta-Analysis

**DOI:** 10.1155/nri/9424887

**Published:** 2025-08-01

**Authors:** Razieh Hajisoltani, Faeze Sadat Ahmadi Tabatabaei, Michael R. Hamblin, Fatemeh Ramezani

**Affiliations:** ^1^Physiology Research Center, Iran University of Medical Sciences, Tehran, Iran; ^2^School of Medicine, Iran University of Medical Sciences, Tehran, Iran; ^3^Laser Research Centre, Faculty of Health Science, University of Johannesburg, Doornfontein 2028, South Africa

**Keywords:** astaxanthin, BBB test, motor performance, neuron, spinal cord injury

## Abstract

**Introduction:** Due to anti-inflammatory, antioxidant, immune-modulating, and antiaging properties of astaxanthin, it has been used to treat spinal cord injuries (SCIs). In this meta-analysis study, the effects of astaxanthin on SCI in animal models were investigated.

**Method:** Scopus, PubMed, Web of Science, and Google Scholar databases were searched based on keywords related to astaxanthin and SCI. The primary screening of articles based on the title and abstract and the secondary screening based on the full text of the articles according to inclusion and exclusion criteria were performed. After extracting the data, statistical analysis was done using STATA software. A standardized mean difference (SMD) was used to analyze the results of the reported studies. Subgroup analysis and quality control of articles was also performed.

**Result:** The overall results showed that astaxanthin has a strong effect (SMD = 3.34; 95% CI: 1.90 to 4.78; *p* < 0.001) on improving motor function after SCI especially when administered in multiple doses over consecutive days. Astaxanthin has a strong effect on reducing lipid peroxidation and increasing antioxidants. Treatment with astaxanthin increased the number of spinal cord neurons and spared white matter.

**Conclusion:** Astaxanthin has the potential to be used as an adjuvant in improving motor behavior, and it is suggested to conduct clinical studies on it.

## 1. Introduction

Spinal cord injury (SCI) is a devastating neurological condition with significant socioeconomic costs to sufferers and the health care system [1]. SCI results in temporary or permanent loss of sensation and motor function below the site of injury, which has a devastating impact on the lives of patients, their families, and society at large [2]. The damage caused by SCI develops in two stages. Direct tissue damage caused by trauma to the spinal cord is the primary stage, followed by biochemical changes marking the secondary stage. The secondary phase of SCI begins with neuroinflammatory responses, followed by an increase in the permeability of the blood–brain barrier, apoptosis of glial and neuronal cells, mitochondrial dysfunction, and oxidative stress. The secondary damage lasts for months and years and leads to sensory-motor dysfunction [3, 4]. Despite some advances in preclinical and clinical studies, there are no effective treatments to reverse the sensorimotor deficits after SCI. Therefore, it is important to find new treatments to target the primary mechanisms involved in SCI for possible clinical application. However, unlike the acute phase, the underlying causes of the secondary effects are not fully understood, and some may be reversible [3, 5, 6]. Among the underlying mechanisms, oxidative stress and inflammation are important. Previous studies have shown that the plasma oxidative stress levels increased following SCI. In addition, plasma levels of inflammatory factors such as tumor necrosis factor-α (TNFα) and other cytokines are increased in individuals with SCI. Therefore, a potent anti-inflammatory drug may be an important therapeutic goal [6–8].

Natural antioxidants have been used as an alternative treatment for SCI. Allicin [9, 10], curcumin [11], quercetin [12, 13], tocotrienols [14, 15], and resveratrol [16, 17] have shown potent ameliorating effects on SCI in preclinical models [18]. It is expected that a potent antioxidant such as astaxanthin will also have such an effect. Astaxanthin is a carotenoid compound found in various living organisms such as marine animals and microorganisms. Astaxanthin has been shown to have a wide range of biological effects, including anti-inflammatory, antioxidant, anticancer, immunomodulatory, and antiaging properties [19, 20]. Astaxanthin has twice as much antioxidant activity as tocopherol or other antioxidant carotenoids [21]. Previous studies have shown that astaxanthin treatment increased the activity of superoxide dismutase (SOD) and catalase (CAT) enzymes, as well as increasing reduced glutathione (GSH) levels. In addition, astaxanthin reduced the levels of malondialdehyde (MDA), a marker of lipid peroxidation, in different parts of the brain [22, 23]. Preliminary research studies have suggested that astaxanthin can modulate immune responses and reduce inflammation [24, 25]. In one in vitro study, astaxanthin was shown to decrease the gene expression of inflammatory mediators, such as interleukin-6 (IL-6), interleukin-1β (IL-1β), and TNF-α in H_2_O_2_-induced cytotoxicity in U937 cells. In addition, astaxanthin has been shown to reduce neuroinflammatory mediators such as TNF-α after SCI in mice [26, 27].

It has been reported that astaxanthin treatment alleviated spinal cord ischemia–reperfusion injury via the activation of the PI3K/Akt/GSK-3β pathway in rats [28]. Astaxanthin also showed significant neuroprotective effects following spinal cord compression injury and reduced neuropathic pain by affecting multiple molecular targets [29, 30]. Astaxanthin could activate the cAMP/PKA/CREB signaling pathway in brain tissues, reduce isoflurane-induced apoptosis by modulating phosphoinositide 3-kinase (PI3K) and its downstream target protein kinase B (Akt), and finally promote the regeneration of axons [30, 31].

In the present work, we conducted a systematic review and meta-analysis study to investigate the therapeutic effects of astaxanthin on restoration of motor function after SCI in preclinical animal models.

## 2. Methods

The current study was designed based on the instructions for conducting a systematic review and meta-analysis (PRISM). We investigated the effect of astaxanthin on restoring motor performance after SCI. Articles were included that investigated the effect of astaxanthin on SCI animal models compared to a SCI group without any treatment and assessed animal movement using the Basso, Beattie, and Bresnahan (BBB) test.

### 2.1. Search Strategy

An extensive search in the electronic databases Medline, ISI Web of Science, EMBASE, CINAHL, and Scopus was conducted [4]. The titles and abstracts of articles, and the word tree in the Mesh and Emtree sections, provided words related to SCI and astaxanthin, which were combined and searched with appropriate tags and Boolean operators. An example of the search strategy designed for the PubMed database is shown in Table 1.

A manual search in Google and Google Scholar was also done to find related articles so that no article was missed. Keywords were selected to be as broad as possible so that no study was excluded. In cases where the data could not be extracted from the article, the authors of that article were contacted.

Short articles and letters to the editor were not included in this study. Review articles and duplicates were not included, but their reference lists were used for manual searching. Studies without a control group and studies that did not measure motor performance were excluded.

### 2.2. Collecting Data

The studies were reviewed and controlled in terms of research methodology by two independent researchers and recorded in the data extraction form, and the relevant reason was recorded in case of rejection. In case of disagreement between the two researchers, a third researcher resolved the disagreement by discussing with the other two researchers. Data collection was done without prejudice or restrictions regarding the authors, journal, organization, or body [4]. The results of the systematic review were recorded in a checklist designed based on the PRISMA statement guidelines. The extracted data include the general information of the article (name of the author and year of publication), the study design, characteristics of the study such as the type of animal, sex and age, the number of samples examined, the dose of administered astaxanthin, the route of administration, the time interval between the injury and the administration of astaxanthin, and the final outcomes. The investigated result was the motor performance of the animals as measured by the BBB test. The latest follow-up time was included in the study. The data reported in the Figures were extracted using Plot Digitizer software. If the data could not be extracted from the article, the corresponding author was asked to provide the data to the researchers. When the evaluated values are presented separately for different subgroups (such as different nanoparticle sizes), the data were recorded separately. When the outcomes to be evaluated were reported in several stages, the latest evaluation time was included. If the results were presented in the form of graphs, data extraction from the graphs was employed.

### 2.3. Statistical Analysis

The results extracted from the articles were recorded as mean and standard deviation and analyzed in STATA 14.0 statistical program. A standardized mean difference (SMD) was calculated for each study, and finally, a pooled effect size was calculated by combining the findings of the studies. Heterogeneity between studies was checked based on the I^2^ test, and based on the presence or absence of heterogeneity, the random effect model or fixed effect model was used to perform the analysis. Subgroup analysis was performed. The funnel plot was used to identify publication bias using Egger's and Begg's tests.

## 3. Results

### 3.1. Screening Articles

**Figure 1:**
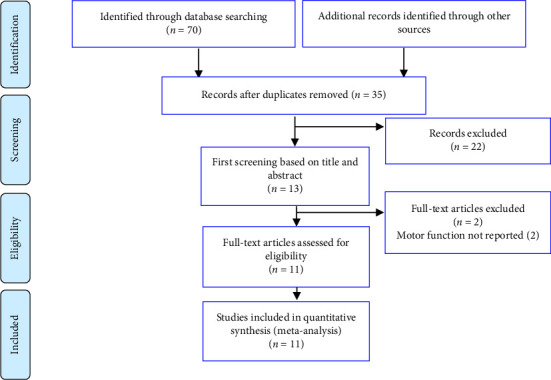
PRISMA plot for the meta-analysis showing database search details, and number of articles included in the study.

From 70 articles, after removing duplicate articles, 35 articles remained. After reading the title and abstract of the article, 13 articles remained in the study. After the full text of the articles was reviewed, 11 remaining articles met the conditions for inclusion in the study (Figure 1).

### 3.2. Data Extraction

Eleven studies were finally included in the meta-analysis that investigated the effect of astaxanthin on the treatment of SCI. In 11 studies, the effect of astaxanthin on the motor function of the animals was investigated. In 5 studies, the effect of treatment with astaxanthin on the number of neurons in the ex vivo spinal cord was investigated. In 4 studies, the effect of treatment on spared white matter was investigated. In 4 studies, the antioxidant effects of astaxanthin on MDA and SOD were investigated. In 3 studies, the effect of astaxanthin on TNF-α expression was investigated. In all the studies, the experiments were performed only on rats (Table 2).

### 3.3. Quality Control

Risk of bias assessment showed that most of the articles were at low risk of bias in terms of species, strain, age/weight, genetic background, number of animals in each group, definition of control, method of allocation to treatments, severity and level of injury, use of appropriate tests, and description of statistical analysis (Table 3).

**Figure 2:**
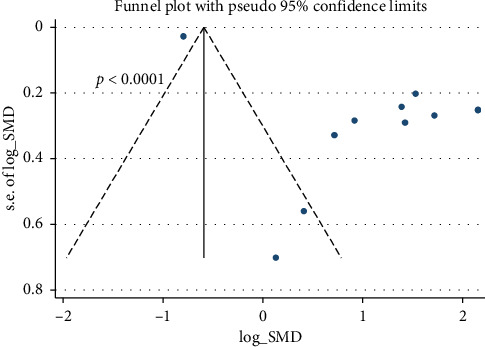
Funnel plot showing the possible occurrence of publication bias between studies that examined motor performance in animals receiving astaxanthin compared with controls.

In the present study, a risk of bias was observed in the 11 trials included in the analysis of the effect of astaxanthin treatment on locomotor activity (*p* < 0.0001) (Figure 2).

### 3.4. Meta-Analysis Results

**Figure 3:**
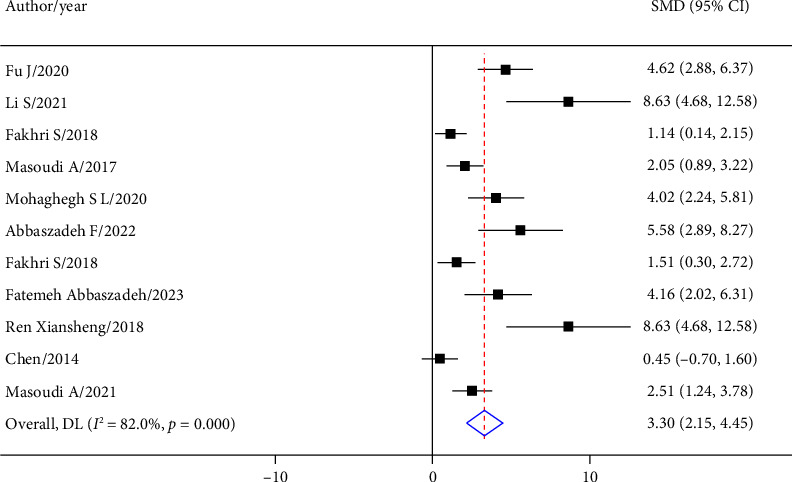
Forest plot of articles investigating the effect of astaxanthin on recovery of movement after SCI with an untreated control group. SMD: standardized mean difference.

In the comparison between the effects of the astaxanthin-treated group and the control group on the improvement of motor function after SCI in 11 separate experiments, the overall results showed that astaxanthin had a strong effect on the improvement of motor performance after SCI (SMD = 3.30; 95% CI: 2.15 to 4.45; *p* < 0.001) (Figure 3).

According to Table 4, the subgroup analysis based on the rat species showed that astaxanthin has a stronger effect on the SD rats (SMD = 5.23; 95% CI: 1.36–9.10; *p*=0.008) compared to the Wistar rats (SMD = 2.65; 95% CI: 1.64–3.63; *p* < 0.0001).

The subgroup analysis based on the injury model showed that there was no difference between the contusion and compression models in the effect of astaxanthin on the improvement of movement behavior after SCI.

With regard to the number of drug administration, in 4 studies, the drug was administered in multidose during several weeks, and in 7 studies, the drug was administered as a single dose. The results show that the effect of astaxanthin injection as a single dose (SMD = 2.65; 95% CI: 1.66–3.63; *p* < 0.0001) compared to the multidose (SMD = 5.23; 95% CI: 1.36–9.10; *p* < 0.001) had a weeker effect. Also, the effect of the administration of astaxanthin intragastrically (SMD = 4.20; 95% CI: 0.13–8.30; *p*=0.043) was more pronounced than with intrathecal administration (SMD = 2.65; 95% CI: 1.66–3.63; *p* < 0.0001).

**Figure 4:**
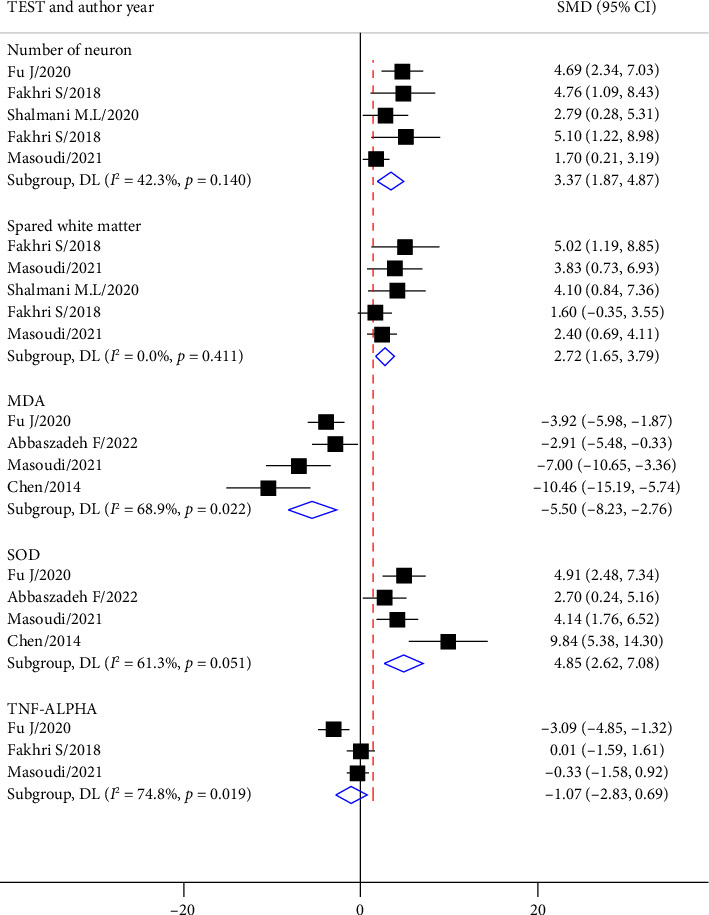
The effects of astaxanthin treatment on the number of neurons, spared white matter, MDA, SOD, and TNFα expression.

In 5 experiments, the effect of treatment with astaxanthin on the number of neurons was investigated, and in 5 experiments, the effect of treatment with astaxanthin on spared white matter was investigated (Figure 4). The results showed that treatment with astaxanthin had a strong effect on increasing the number of neurons (SMD = 3.37; 95% CI: 1.87 to 4.87; *p* < 0.0001) as well as increasing the spared white matter (SMD = 2.72; 95% CI: 1.65 to 3.79; *p* < 0.0001) (Figure 4).

The results from 3 studies showed that astaxanthin had a strong effect on reducing the expression of MDA (SMD = −5.50; 95% CI: −8.23 to −2.76; *p* < 0.0001), and the results from 4 studies showed that astaxanthin had a strong effect on increasing the expression of SOD (SMD = 4.85; 95% CI: 2.62 to 7.08; *p* < 0.0001). The effect of astaxanthin on the level of TNF-α expression from 3 studies was not significant (SMD = 1.071; 95% CI: −2.828–0.686; *p*=0.23) (Figure 4).

## 4. Discussion

In this meta-analysis study, we compared the effect of astaxanthin treatment on the motor performance of animals with SCI with that of control SCI animals that did not receive any treatment. The overall results showed that astaxanthin had a strong effect on improving motor performance after SCI alongside with antioxidant effect. The overall results showed that astaxanthin has a strong antioxidant effect after SCI and increases the number of neurons and the spread white matter, which ultimately leads to a strong effect of astaxanthin on improving motor function after SCI.

SCI is a neurological condition characterized by sensory and motor disabilities, accompanied by the activation of oxidative stress, apoptosis, and autophagy pathways. From a mechanistic perspective, SCI increases the generation of reactive oxygen species (ROS), which cause oxidative stress and inflammatory responses, ultimately leading to neuronal apoptosis/autophagy and irreversible neuronal damage. The response to mild oxidative stress is a natural protective mechanism of the body that plays a role in regulating processes such as cell signal transduction, cell proliferation, and apoptosis [42]. Mitochondrial dysfunction is an important factor leading to neuronal cell death following SCI, which is directly related to a marked accumulation of calcium inside the damaged cells as well as with increased oxidative stress [43]. Oxidative stress following SCI disturbs the ionic homeostasis inside and outside the neuronal cell membranes, and a large amount of calcium ions enter the mitochondria and accumulate, causing damage to the mitochondria, and they ultimately inhibit ATP synthesis. Studies on SCI animal models have shown that following the initial injury, the MDA content in the spinal cord was increased, while there was a significant decrease in the total antioxidant capacity (TAC) and the expression levels of SOD and glutathione peroxidase (GPx). These changes suggest that SCI can produce damage to the spinal cord tissues by triggering a pronounced increase in oxidative stress.

Therefore, treatment with neuroprotective or antioxidant agents could reduce oxidative stress and conceivably lead to a reduction in neuronal damage. Astaxanthin, also known as the crab shell pigment, is a pink-colored carotenoid compound, chemically similar to beta-carotene. Studies have shown that astaxanthin has powerful anti-inflammatory, antioxidant, antitumor, and other pharmacological properties [44, 45]. In one study conducted by Lou and colleagues, in 2014, it was shown that astaxanthin effectively reduced the concentration of MDA and increased the concentrations of SOD and CAT in SCI model rats. Moreover, their results showed that astaxanthin increased the activity of antioxidant enzymes while reducing oxygen free radicals and lipid peroxidation. In another study conducted by Salamoto and colleagues, it was found that astaxanthin had antioxidative properties and significantly increased the levels of SOD, CAT, GSH-PX, and GSH in diabetic rats [46]. The results of the present study showed that astaxanthin lowered MDA expression (in three articles) and increased SOD expression (in three articles) confirming that astaxanthin had a strong antioxidant effect in SCI rats.

Locomotor dysfunction is one of the main symptoms that occur after SCI, which is mainly caused by axonal damage and the loss of motor neurons and oligodendrocytes. Therefore, treatment with neuroprotective agents to restore motor function has always been an attractive subject to be investigated in SCI studies. Studies have shown that astaxanthin has outstanding neuroprotective effects in ischemic and hemorrhagic stroke models in rodents [47]. For example, in a subarachnoid hemorrhage model in mice, it was shown that the administration of astaxanthin at 30 min after the injury could significantly reduce neuronal cell death and improve neuronal dysfunction. Evidence has also shown that astaxanthin treatment has neuroprotective effects in animal models of traumatic brain injury. It was demonstrated that treatment with astaxanthin improved sensorimotor function and cognitive function in a rat model of brain injury [48]. Furthermore, Fakhri and colleagues evaluated the effect of astaxanthin treatment in a SCI animal model. Their results showed that the administration of astaxanthin enhanced motor performance in SCI. They also showed that treatment with astaxanthin significantly reduced the loss of neurons and demyelination in the central part of the lesion.

One new report suggested that astaxanthin could effectively protect neurons from apoptosis after brain injury, thereby preserving neuronal function [49]. Wu and colleagues found that treatment with astaxanthin improved brain function by activating nuclear factor erythroid 2-related factor 2 (NRF2), increasing antioxidant response pathways, reducing brain edema, BBB damage, and inhibiting cell apoptosis in a mouse model of subarachnoid hemorrhage [30]. Additionally, astaxanthin significantly increased the level of phosphorylated Akt and Bad in the cerebral cortex and significantly decreased the level of caspase 3, thereby exerting protective effects in the brain [30]. Wang and colleagues investigated a brain injury model produced by isoflurane and showed that astaxanthin inactivated the caspase 3 pathway, protected the cells from apoptosis, increased cell proliferation, and activated the phosphatidylinositol signaling pathway 3-kinase to improve neuronal function [50].

In addition, astaxanthin could play an important role in neuroprotection by inhibiting nuclear factor NF-kB signaling and caspase-3 activity, reducing the expression of proinflammatory cytokines, inhibiting the sodium-potassium-chloride cotransporter, and reducing Bax levels. At the same time, astaxanthin increased the level of Bcl-2 which helped to reduce apoptosis in astrocytes [51]. In a study conducted by Pan, it was discovered that astaxanthin enhanced antioxidant capacity, triggered antioxidant defense pathways, inhibited ROS and apoptosis, and promoted nerve regeneration in an ischemic stroke model in rats. Astaxanthin reduced the volume of the cerebral infarction area and enhanced neurological function [52].

Studies in SCI models have shown that demyelination and loss of neurons occur in the rostral and caudal regions of the injured spinal cord and that treatment with astaxanthin protected the neurons against this damage [36, 53].

In a study conducted by Abbas Zadeh, it was found that autophagy occurred between the first and seventh days following SCI. Additionally, it was found that treatment with astaxanthin inhibited this process and reduced histopathological changes as well as neural degeneration in spinal cord tissue [36]. The results of this study showed that astaxanthin significantly reduced spinal cord edema, lessened the histopathological and ultrastructural damage to the spinal cord, and protected spinal cord tissue and motor function in rats with SCI.

Regarding the number of times astaxanthin was administered, the drug was administered in multidose in several consecutive days which has a more strong effect compared to once administration.

The subgroup study revealed that administering astaxanthin intragastrically yielded more potent effects than intrathecal administration. The variability in astaxanthin dosing, administration routes, and treatment durations across studies, as well as the small number of studies in each subgroup, are mandatory limitations of the present study. For example, the number of studies that used rats (4 studies), the number of studies that used the compression model (4 studies), the number of multiple-dose studies (4 studies), and intragastric administration (3 studies) are small. This small number of studies in each group has limited the generalizability of the results, and there is a need for more studies with these characteristics.

According to the results of the present study, astaxanthin has no significant effect on the expression of TNFα. However, this result is the result of the analysis of data obtained from 3 studies, and this result should be expressed with great caution, and further study is needed to investigate the effect of astaxanthin on TNF expression after SCI.

The results of a meta-analysis study conducted by Long-Yun Zhou and colleagues in 2023, which analyzed data from 5 studies, showed that astaxanthin had a very strong effect on improving motor behavior after SCI, which is consistent with the results of our study with 11 included studies [54].

Considering that astaxanthin is a common dietary supplement widely available in health food stores, given the results of the present meta-analysis based on preclinical studies, we believe that a clinical trial of the oral administration of astaxanthin in patients with acute SCI should be given more attention. The results of a clinical study investigating the effect of astaxanthin on SCI patients have not been reported yet. It is recommended that a clinical trial study is conducted on SCI patients in the acute or subacute phases and , after few months, motor function, spasm, muscle activity, tissue damage, and serum levels of inflammatory factors be compared between a patient group that received medication and a control group that did not.

## Figures and Tables

**Table 1:** Search strategy designed for PubMed database.

(“Astaxanthin” [TIAB]) and (“spinal cord injury” [MeSH] OR “spinal cord contusion” [MeSH] OR “spinal cord hemisection” [MeSH] OR “spinal cord transsection” [MeSH] OR “cervical spine injury” [MeSH] OR “spinal cord injury” [TIAB] OR “spinal cord contusion” [TIAB] OR “spinal cord hemisection” [TIAB] OR “spinal cord transsection” [TIAB] OR “cervical spine injury” [TIAB] OR “spinal compression” [TIAB] OR “spinal cord trauma” [TIAB] OR “trauma, spinal cord” [TIAB] OR “injured spinal cord” [TIAB] OR “spinal cord injured” [TIAB] OR “spinal cord injuries” [TIAB] OR “nerve transection” [TIAB])

**Table 2:** The data of the studies available in the meta-analysis that have been examined in these articles are the effect of astaxanthin on the spinal cord injury.

Author, year	Gender/species/strain/weight or age	Animals per group (SCI/treat)	SCI model/injury location/severity of injury/injury to treatment interval (days)	Protocol of treatment	Administration route	FU	Outcomes
Fu et al. (2020) [27]	Male/Sprague-Dawley/rats/180–200 g	10/10	Ischemia–reperfusion contusion injury/NR/severe/14 days before surgery	14 days before SCI, daily	Intragastric	72 h	Movement score, number of neurons, MDA, SOD, IL-IB, TNF-α
Li et al. (2021) [32]	Male/female/Sprague-Dawley/rats/280 ± 20 g	6/6	Modified Allen method/T10/NR/immediately after surgery	Twice per day until the end of experiment	Intragastric	4 Weeks	Movement score, number of neurons, MDA, SOD
Fakhri et al. (2018) [33]	Male/Wistar/rat/30–260 g	9/9	Compression/T8-T9/moderate/30 min after surgery	Once	Intrathecal	4 weeks	Movement score, number of neurons, spared white matter
Masoudi et al. (2017) [34]	Male/Wistar/rat/250–280 g	9/9	Contusion/T8-T9/severe/30 min after surgery	Once	Intrathecal	4 weeks	Movement score, spared white matter
Mohaghegh et al. (2020) [35]	Male/Wistar/rat/250–300 g	8/8	Contusion/T8-T9/severe/30 min after surgery	Once	Intrathecal	6 weeks	Movement score, number of neurons, spared white matter
Abbaszadeh et al. (2022) [36]	Male/Wistar/rat/230–250 g	6/6	Compression/T8-T9/moderate/30 min after surgery	Once	Intrathecal	1 week	Movement score, MDA, SOD
Fakhri et al. (2018) [37]	Male/Wistar/rat/230–270 g	7/7	Compression/T8-T9/severe/30 min after surgery	Once	Intrathecal	4 weeks	Movement score
Abbaszadeh et al. (2023) [37, 38]	Male/Wistar/rat/230–250 g	6/6	Compression/T8-T10/moderate/30 min after surgery	Once	Intrathecal	4 weeks	Movement score
Xiansheng et al. (2018) [39]	Both//SD/rat/280 ± 20g	6/6	Contusion/T9-T11/immediately after surgery	Twice a day until the end of experiment	Gavage	4 weeks	Movement score
Chen et al. (2014) [40]	Female/SD/rat/260–300 g	6/6	Contusion/T12/5 min after surgery	Once	Intragastric	3 weeks	Movement score, MDA, SOD
Masoudi et al. (2021) [41]	Male/Wistar/rat/250–280 g	9/9	Contusion/T8-T9/severe/30 min after surgery	Once	Intrathecal	4 weeks	Movement score, number of neurons, spared white matter

Abbreviations: FU, follow-up; NR, not reported.

**Table 3:** All the articles were at risk of bias in terms of description of the reasons to exclude animals from the experiment during the study.

Ref	1	2	3	4	5	6	7	8	9	10	11	12	13	14	15	% of bias
Fu et al. [27]	L	L	L	L	L	L	L	L	H	H	L	L	L	L	H	20
Li et al. [32]	L	L	L	L	L	L	L	L	L	L	L	L	L	L	H	6.6
Fakhri et al. [33]	L	L	L	L	L	L	L	L	L	L	L	L	L	L	H	6.6
Masoudi et al. [34]	L	L	L	L	L	L	L	L	L	L	L	L	L	L	H	6.6
Mohaghegh et al. [35]	L	L	L	L	L	L	L	L	L	L	L	L	L	L	H	6.6
Abbaszadeh et al. [36]	L	L	L	L	L	L	L	L	L	L	L	L	L	L	H	6.6
Fakhri et al. [37]	L	L	L	L	L	L	L	L	L	L	L	L	L	L	H	6.6
Abbaszadeh et al. [38]	L	L	L	L	L	L	L	L	L	L	L	L	L	L	H	6.6
Xiansheng et al. [39]	L	L	L	L	L	L	L	L	L	H	L	H	L	H	H	20
Chen et al. [40]	L	L	L	L	L	L	L	L	L	H	L	H	L	H	H	26.6
Masoudi et al. [41]	L	L	L	L	L	L	L	L	L	L	L	H	L	L	H	13.3
	0%	0%	0%	0%	0%	0%	0%	0%	9%	27.2%	0%	27.2%	0%	18.1%	100%	

*Note:* The article by Chen had the highest risk of bias with a 26.6% overall risk (Table 3). Quality control of included articles in the study. Items: 1. Species; 2. designation of strain; 3. age/weight; 4. genetic background; 5. number of animals per group; 6. definition of control; 7. method of allocation to treatments; 8. severity of injury; 9. level of injury; 10. bladder expression; 11. using appropriate tests; 12. blinding of assessor; 13. description of statistical analysis; 14. regulation and ethics; 15. description of the reasons to exclude animals from the experiment during the study (attrition). L: low risk; H: high risk.

**Table 4:** Subgroup analysis of the effect of astaxanthin treatment in improving motor performance after spinal cord injury compared to nontreated animals.

Subgroup	Number of experiments	Heterogeneity (*p* value)	SMD (95% CI)	*p* value
*Rat strain*				
Wistar	7	68.5% (0.004)	2.65 (1.64–3.63)	*p* < 0.0001
Sprague-Dawley	4	91.8% (0.000)	5.23 (1.36–9.10)	*p*=0.008

*SCI model*				
Contusion	7	84.9% (0.000)	3.75 (2.09–5.41)	*p* < 0.0001
Compression	4	78.6% (0.003)	2.73 (1.03–4.44)	*p*=0.002

*The number of times to receive the drug*				
Single dose	7	68.5% (< 0.0001)	2.65 (1.66–3.63)	*p* < 0.0001
Multidose	4	91.8% (*p*=0.06)	5.23 (1.36–9.10)	*p*=0.008

*Administration rout*				
Intragastric	3	92.4% (< 0.0001)	4.20 (0.13–8.30)	*p*=0.043
Intrathecal	7	68.5% (*p*=0.004)	2.65 (1.66–3.63)	*p* < 0.0001
Intravenous	1	—	—	—

Overall	11	91.9% (< 0.0001)	3.30 (2.15–4.45)	*p* < 0.0001

## Data Availability

The data that support the findings of this study are available from the corresponding author upon reasonable request.
